# A Magnetoelectrochemical Bioassay for Highly Sensitive Sensing of Point Mutations in Interleukin-6 Gene Using TMB as a Hybridization Intercalation Indicator

**DOI:** 10.3390/bios13020240

**Published:** 2023-02-08

**Authors:** Sabrine Baachaoui, Mohamed Mastouri, Maroua Meftah, Basma Yaacoubi-Loueslati, Noureddine Raouafi

**Affiliations:** 1 Sensors and Biosensors Group, Laboratory of Analytical Chemistry and Electrochemistry (LR99ES15), Chemistry Department, Faculty of Science, University of Tunis El Manar, Tunis 2092, Tunisia; 2 Laboratory of Mycology, Pathologies and Biomarkers (LR16ES15), Biology Department, Faculty of Science, University of Tunis El Manar, Tunis 2092, Tunisia

**Keywords:** point mutation, human DNA, sensing, electrochemistry, cancer, interleukin-6, ovarian cancer

## Abstract

Point mutations are common in the human DNA genome and are closely related to higher susceptibility to cancer diseases. Therefore, suitable methods for their sensing are of general interest. In this work, we report on a magnetic electrochemical bioassay using DNA probes tethered to streptavidin magnetic beads (strep-MBs) to detect T > G single nucleotide polymorphism (SNP) within the inteleukin-6 (IL6) gene in human genomic DNA. In the presence of the target DNA fragment and tetramethylbenzidine (TMB), the electrochemical signal related to the oxidation of TMB is observed, which is much higher than the one obtained in the absence of the target. The key parameters affecting the analytical signal, such as the concentration of the biotinylated probe, its incubation time with strep-MBs, DNA hybridization time, and TMB loading, were optimized using the electrochemical signal intensity and signal-to-blank (S/B) ratio as selection criteria. Using spiked buffer solutions, the bioassay can detect the mutated allele in a wide range of concentrations (over six decades) with a low detection limit (7.3 fM). Furthermore, the bioassay displays a high specificity with high concentrations of the major allele (one mismatched), and two mismatched and non–complementary DNA. More importantly, the bioassay can detect the variation in scarcely diluted human DNA, collected from 23 donors, and can reliably distinguish between heterozygous (TG genotype) and homozygous (GG genotype) in respect to the control subjects (TT genotype), where the differences are statistically highly significant (*p*-value < 0.001). Thus, the bioassay is useful for cohort studies targeting one or more mutations in human DNA.

## 1. Introduction

Benzene, strong oxidizing agents, and some mycotoxins are a few examples of environmental carcinogens; these, as well as personal habits such as heavy smoking and alcohol abuse, can induce somatic DNA mutations, which may develop into a form of cancer if the mutations are not repaired by DNA repairing tools [1]. Furthermore, point mutations can increase the susceptibility to cancer [2], while some mutations are known to be protective against cancer [3]. For instance, BRCA1 and BRCA2 are two tumor suppressor genes [4]. SNPs in these genes are responsible for 55–72% and 45–69%, respectively, of the inherited breast cancer cases [5]. Yaacoubi-Loueslati and coworkers recently showed that point mutations (namely rs1880269 and rs1800469) in IL6 and TGFβ1 genes have a protective role in ovarian cancer [6]. Moreover, genetic variations in other genes were also associated to a higher susceptibility of ovarian cancer [3].

Fluorescence [7,8], surface plasmon resonance [9], or electrochemistry [10] are routinely used to detect point mutations in human or bacterial DNA. The procedure can be either used to detect short sequences with a high similarity such as miRNAs [11,12,13,14,15,16,17,18,19,20,21,22] or dsDNA [10,23]. On the other hand, small organic molecules, which can intercalate between DNA bases such as methylene blue (MB) [24], tetramethylbenzidine [25], doxorubicin [26], acridine derivatives [27], Hoechst 33258 [28], anthraquninone-2-sulfonic acid [29], etc. [30] are used for electrochemical transduction of the recognition event between the probe and target DNA strands. For example, Jin et al. used methylene blue for the sensing of DNA based on the use of a hairpin probe [31]. Scalpel enzyme implemented the detection of a single nucleotide polymorphism in PRSS1 gene; the amount of intercalated MB was assessed by coulometry [32]. Alves-Balvedi et al. employed TMB as an intercalation redox marker to follow the DNA hybridization and applied the concept for the sensing of the Epstein–Barr virus [25].

Several strategies to detect short sequences of RNA or DNA used modified MBs and enzyme-assisted amplification [17]. Because of their wide spread in the human genome, several methods have been designed to detect point mutations in DNA [33]. For instance, point mutations in DNA can be also electrochemically probed using (amp)ligase-assisted DNA ligation [34], streptavidin-HRP amplification [35], molecular beacons [36], rolling circle amplification [37], ferrocene-modified gold nanoparticles [38], redox markers such as naphthoquinone tethered to the electrode surface [23], etc. In one example, Meftah et al. utilized two hairpin probes immobilized on the electrode surface to electrochemically detect two SNPs in IL6 and TGFβ1 genes using methylene blue as a redox probe [39].

In this work, we report on the use of DNA-modified magnetic beads to detect short sequences of human DNA using TMB as a redox intercalating agent. Under optimal conditions, the bioassay can detect the mutated allele in a wide range of concentrations with a low detection limit and a total discrimination towards wild and noncomplementary sequences. Interestingly, the bioassay was used to detect genetic variations in human DNA samples, collected from 23 donors, and can reliably distinguish GT and GG genotypes from the ancestral TT genotype.

## 2. Materials and Methods

### 2.1. Chemicals and Solutions

All the reagents used are high analytical grade and purchased from Sigma–Aldrich (Taufkirchen, Germany): Tris–HCl, 99%; EDTA, 99%; ready TMB solution for ELISA; sulfuric acid (>98%); and RNA-free water (18 MΩ cm at 25 °C). Streptavidin-coated magnetic beads (4 mg/mL) were purchased from New England Biolabs (Frankfurt am Main, Germany). All the oligonucleotides were synthetized and purified by HPLC by Carthagenomics (Tunis, Tunisia). Table 1 summarizes the sequences of all the used synthetic oligonucleotide DNA. Aliquots were prepared in RNA-free water and stored at −20 °C.

### 2.2. Sample Collection and Genotyping

The real samples were gathered from controls and patients recruited at the Salah Azaiez Oncology Institute (ISA, Tunis, Tunisia). The study was approved by the ISA ethical committee (ref. ISA/2019/01). The DNA was extracted from blood using QIAamp DNA blood mini kit, according to the manufacturer’s recommendations (Qiagen GmbH, Hilden, Germany).

### 2.3. Electrochemical Measurements

Prior to use, the electrode was cleaned by cyclic voltammetry from –1.0 V to +1.0 V vs. Ag/AgCl/KCl using 1 M sulfuric acid solution. After each reading, the electrode was cleaned with H_2_SO_4_ (1 M) solution to remove adsorbed TMB molecules; then, cyclic voltammetry was used to check the electrode surface using Fe(CN)_6_]^3/4−^ (5.0 mM) as a redox probe.

For the electrochemical measurements, we used square wave voltammetry (SWV) to oxidize the intercalated TMB into the double-stranded DNA. The potential was scanned from +0.1 V to +0.6 V vs. Ag/AgCl/KCl. The optimized parameters for recording the SWV curves were: potential step of 5 mV, potential amplitude of 50 mV, and frequency of 10 Hz. The currents were collected at the peak maximum at *ca*. +0.25 V vs. Ag/AgCl/KCl (3 M).

### 2.4. Probe Assembly, DNA Hybridization, and TMB Intercalation

An amount of 5 µL of the commercial streptavidin-coated MBs (4.0 mg/mL) solution was washed once by 50 µL of the W&B buffer (20 mM Tris-HCl, 0.5 M NaCl, 1.0 mM EDTA at pH 7.5), then 10 µL of 5 µM of the biotinylated pIL6 probe and 40 µL of the W&B buffer were added, and the mixture was incubated for 30 min at 37 °C. After incubation, the beads were magnetically separated and washed three times using 50 µL of the SSCI buffer (0.75 M NaCl, 0.075 M sodium citrate) to remove the excess of the unreacted probe.

An amount of 10 µL of the desired amount of the target DNA was added to the pIL6-modified MBs, followed by 40 µL of SSCI buffer. The obtained solution was incubated for 45 min at 37 °C. After magnetic separation, the beads were washed three times by 50 µL of the SSCI buffer to remove the excess of the target.

For the specificity study, each sequence was tested individually using the same procedure as for the tIL6 sequence. In brief, 10 µL of 5.0 nM of tIL6–1m, tIL6–2m or the noncomplementary sequence, and 40 µL of the SSCI buffer were added to the probe-modified MBs and incubated at 37 °C for 45 min. The beads were magnetically separated from the excess of the target DNA and washed with buffer.

An amount of 50 µL of TMB solution (diluted 3×) was added to the dsDNA/MBs and incubated for 15 min (37 °C), after magnetic separation and washing three times with PBS solution (10 mM, pH 7.4) to remove the excess of TMB. The electrochemical signal was recorded using SWV.

For the control experiments, all the steps were the same but without the addition of the target DNA sequence. All the experiments were run in triplicate and the relative standard deviation was estimated from the recorded data.

### 2.5. Detection of Genomic DNA

Before reading, the genomic DNA samples were treated according to previous reports [39,40]. In brief, 5 µL of genomic DNA (10 µg·mL^−1^) was diluted five times with the EcoRI buffer, then 10 µL of EcoRI (0.1 U·µL^−1^) was added, and the mixture was incubated for 30 min. An amount of 10 µL of the resulting solution was heated to 95 °C for 5 min, to unwind the dsDNA, and was then added to the MBs/pIL6 and incubated for 45 min at 37 °C. After magnetic separation and washing, 50 µL of TMB solution and the solution was incubated for 15 min (37 °C). After the last magnetic separation step and washing with PBS solution, the electrochemical oxidation signal of TMB was recorded using SWV.

## 3. Results

### 3.1. Design of the Bioplatform

**Scheme 1:**
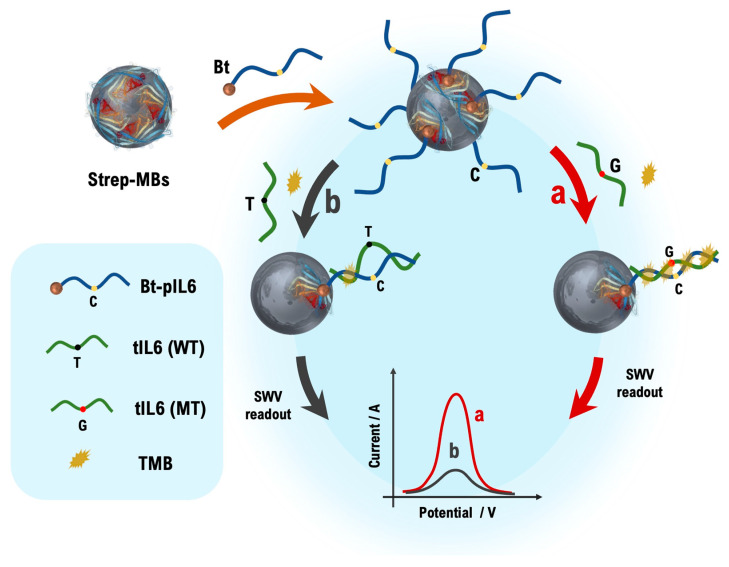
Schematic illustration of the magnetic electrochemical bioassay for the sensing of the single point mutation in genomic IL6 DNA. (a) The probe fully recognizes the mutated target and after TMB intercalation, a strong oxidation signal is obtained and (b) the probe partially recognizes the wild target and a weak redox signal is observed.

Scheme 1 depicts the different steps of the magnetic electrochemical bioassay in detecting the target or genomic DNA. In the first step, the biotinylated probes were tethered to the magnetic beads surface thanks to the high affinity of streptavidin to biotin present at the 5′- end of the DNA probes. Once the bioconjugation achieved, the pIL6/MBs were hybridized with the synthetic target sequence (21 nt) containing the SNP or with the scarcely treated genomic DNA fragment. Finally, TMB was intercalated into the double-stranded DNA formed between the DNA probe and the DNA target. TMB is widely used as an electron donor in the ELISA assay but not well exploited in DNA sensors although it is a good DNA intercalator [25]. Among the goals of this investigation is the aim to extend the use of TMB as a probe in DNA biosensors.

**Figure 1:**
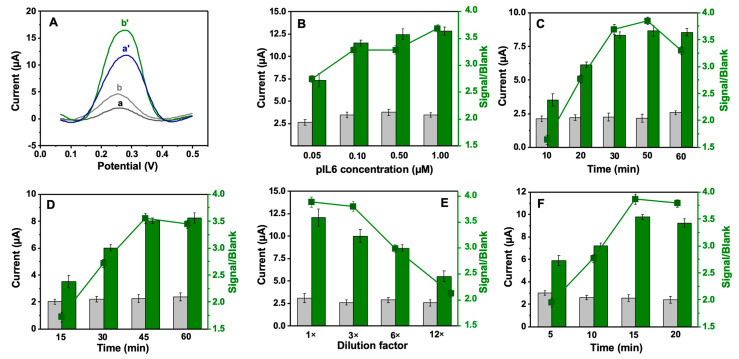
(**A**) SWV responses obtained in presence of the synthetic tIL6 sequence using MB (a and a’) and TMB (b and b’). Current and S/B ratio variation as function of the (**B**) pIL6 concentration, (**C**) incubation time of pIL6, (**D**) hybridization time of tIL6 sequence with pIL6/MBs, (**E**) dilution ratios of commercial TMB, and (**F**) incubation time into TMB solution. The 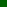 symbol represents the signal-to-blank ratio values for each measurement.

Preliminary results showed that using the raw DNA extract yielded a very high current response due to the intercalation of TMB into the long DNA double strand. Treatment of the DNA with a DNA scalpel enzyme such as EcoR I is mandatory to obtain useful results. Indeed, the enzyme allowed the cleaving of the genomic DNA into small fragments of 400–500 bp [40,41]. We also examined the use of methylene blue as a redox intercalation agent; we found that the TMB obtains a higher redox signal. We therefore chose to carry out the work with TMB (Figure 1A).

### 3.2. Optimization of the Readout Parameters

To obtain the highest analytical signal, we optimized all the relevant parameters involved in the bioassay (Figure 1B–F). For a better accuracy, we used as selection criteria both the current intensity and the signal-to-blank (S/B) ratio [11,12,13,15,16,17,18]. First, we looked at the biotinylated pIL6 probe concentration tethered to the streptavidin-coated magnetic beads. This influences the electrochemical signal by controlling the amount of the hybridized target DNA and, consequently, the amount of intercalated TMB molecules. Figure 1B shows that both the current and the S/B ratio increase concomitantly with the pIL6 probe concentration until 1.00 µM; we did not test higher concentrations of the DNA probe. We selected this probe concentration to carry out further work. Incubation time of the pIL6 probe was also assayed. We examined incubation time varying from 10 min to 60 min. Results showed that, over 30 min, the signal remained almost unchanged (Figure 1C). We chose this duration to shorten the bioassay time. Furthermore, we investigated the hybridization time between the tIL6 target sequence and the pIL6-modified magnetic beads; times from 15 min to 60 min were examined, and the best current and S/B were observed for 45 min incubation at 37 °C. We chose this time to carry out further work (Figure 1D).

Different dilutions of the ready-to-use TMB solution ranging from undiluted (1×) to 12× were also examined (Figure 1E). We found that higher TMB concentrations (i.e., 1× to 3× dilutions) provide a high analytical signal than 12×. We chose to use 3× diluted TMB solution for further work. In the last step, we studied the incubation time of the tIL6/pIL6/MBs bioconjugate into the diluted TMB solution for durations ranging from 5 min to 20 min (Figure 1F). We found that 15 min is enough to yield a good electrochemical signal even for very low concentrations of the target sequence. This result is in a good agreement with the literature. Indeed, Meftah et al. used an optimized time of 15 min to reveal the DNA recognition with hairpin-DNA-modified screen-printed electrodes [39], and Raouafi et al. incubated a bioelectrode into an MB solution for 30 min in order to intercalate the redox probe into the DNA [24]. The investigated intervals and the optimal values are summarized in Table 2.

### 3.3. Analytical Characteristics

#### 3.3.1. Dynamic Range and Detection Limits

**Figure 2:**
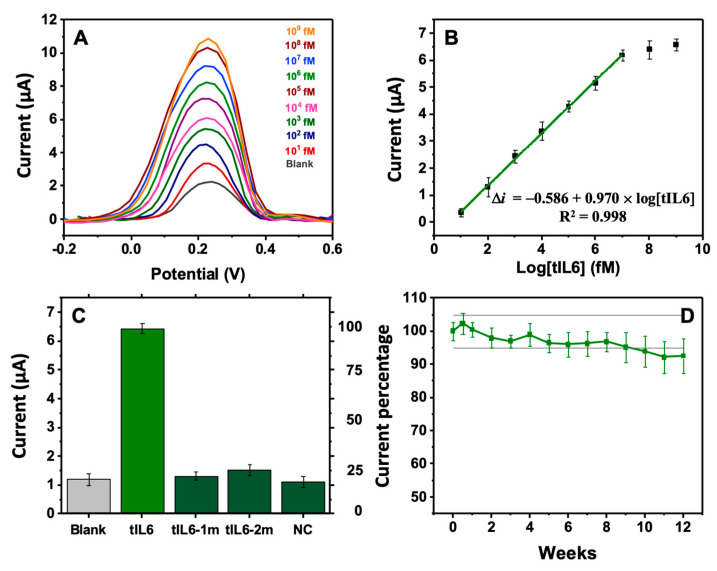
(**A**) SWV curves of pIL6/MBs in presence of increasing concentrations of the tIL6 sequence. (**B**) Calibration plot for the tIL6 sequence showing the dynamic range equation, (**C**) currents and percentages obtained with 500 pM of tIL6, or 5.0 nM of tIL6-1m, tIL6-2m, or the noncomplementary sequence compared with the blank response, and (**D**) stability response of the bioconjugate assayed over a period of three month.

To assess the analytical performances of the bioassay, the pIL6/MBs were incubated into spiked solutions of the mutated tIL6 sequence (minor allele), where the range of concentrations varies from 10 fM to 10^9^ fM. After magnetic separation and washing, the tIL6/pIL6/MBs bioconjugate was incubated into a diluted TMB solution for 15 min. After a final magnetic separation and washing step, the square-wave voltammetry curves were recorded at the optimal parameters. SVW curves are provided in Figure 2A. The results showed that the current differences, Δ*i*, measured before and after the incubation in the tIL6 solutions, increased along the increase of the target concentrations. The plot of the current difference versus the tIL6 concentrations is linear according to the equation: Δ*i* = −0.586 + 0.970 × log[tIL6] (R^2^ = 0.998) (Figure 2B). Furthermore, the linear range spanned over six decades of concentrations in a logarithmic scale and the detection limit was evaluated to be 7.3 fM, calculated as three times the ratio of the current deviation for ten different bioassays divided by the slope of the calibration curve.

#### 3.3.2. Specificity and Stability

To investigate the specificity of the bioassay, the pIL6/MBs were challenged with the unmutated sequence, which has one point mutation at the middle of the nucleic sequence, at a concentration 10 times higher than that of the target sequence (Figure 2C). The normalized response does not exceed 24% of that observed with a much lower concentration of the target. Likewise, the voltamperometric responses were recorded in the presence of two mismatched or the noncomplementary sequence; each of the amperometric responses was similar to the blank response. The current percentages are less than 25% for both sequences, suggesting a high specificity of the bioassay.

The pIL6/MBs bioconjugate, prepared and stored without removing the excess of the biotinylated probe, was assayed on a weekly basis for three months in presence of 500 pM of the target sequence. Prior to use, the magnetic beads were magnetically separated from the unbound sequences and washed with the W&B buffer. As Figure 2D shows, for the normalized responses, the bioassay maintained more than 95% of the initial response for almost nine weeks; probably, storing the pIL6-conjuated magnetic beads in the presence of the excess the bt-pIL6 DNA prevented the desorption of the bt-pIL6 due the absence of a concentration gradient.

#### 3.3.3. Comparison with the Literature

Table 3 gathers several examples of electrochemical genosensors to detect SNPs in DNA. The set can be divided into biosensors using enzymes for electrochemical signal amplification and others without signal amplification. Enzymes with high turnover rates, such as glucose oxidase (GOx), alkaline phosphatase (ALP), or horseradish peroxidase (HRP), were used for signal amplification. The electrochemical transduction methods were amperometry (i-t), differential pulse voltammetry (DPV), or SWV.

This bioassay presents a wider dynamic range with a low detection limit than those without enzymatic signal amplification using the redox signals generated from curcumin [38], naphthoquinone [23], methylene blue [39], and guanine [47]. Furthermore, the dynamic range and the detection limit are better, in some cases, than those using glucose oxidase [42] and alkaline phosphatase [43,44] for signal amplification. The performances are close to those using a double-labeled probe with biotin and digoxigenin tethered to a gold electrode array [45] and outperformed by the device used for the detection of lacZ gene using a streptavidin-HRP chain and TTF-modified gold screen-printed electrodes [46]. One of the major advantages of the bioassay is the use of MBs that can be readily recovered using magnetic separation to capture the target sequence; however, the performance of MBs could be significantly affected by surface modifications.

### 3.4. Real Sample Analysis

**Figure 3:**
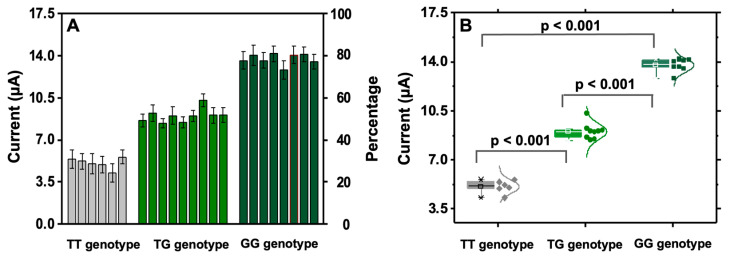
Amperometric response from the analysis (**A**) and (**B**) box plot showing the currents and their mean values recorded for real samples of control subjects and patients (diamonds, circles, and squares) having heterozygous or homozygous genotypes in IL6. One-sample *t*-test statistical analysis *p*-value is <0.001.

Next, we analyzed 23 real samples of genomic DNA voluntarily obtained from 6 control subjects and 17 ovarian cancer patients. The controls had the major allele DNA belonging to TT genotype; the ovarian cancer patients were either heterozygous (TG genotype) or homozygous (GG genotype) according to the biomolecular genotyping by real-time PCR analysis. Figure 3A shows the current traces obtained in the presence of the scarcely treated human DNA. Overall, the current responses with the mutated DNA sequences had higher intensities compared with the unmutated DNA. Furthermore, the DNA samples of the homozygous patients, having two point mutations in both alleles, even provided higher currents. The data show that the bioassay can differentiate heterozygous from homozygous patients since the method is sensitive to the amount of the minor allele DNA. Statistical analysis using the one-sample *t*-test method performed for the three sets of samples related to the controls and the heterozygous, the controls and the homozygous, and the heterozygous and the homozygous each time the obtained a *p*-value is less than 0.001, indicating that the differences can be considered as statistically highly significant (Figure 3B).

## 4. Discussion

The work reports on the design of a magnetic electrochemical bioassay to detect rs1880242 single nucleotide polymorphism occurring in human IL6 gene. This SNP was recently demonstrated by Ben-Ahmed et al. to be positively linked to the susceptibility of women to ovarian cancer, using a cohort study involving 70 cases [6]. The bioassay stepwise was easy to carry out using commercial reagents and the method applied to the sensing of point mutations in spiked buffer solutions and scarcely treated genomic DNA samples. The overall process of the bioassay was straightforward, starting with conjugating streptavidin-coated MBs with biotinylated DNA sequences. The bioconjugate was subsequently incubated in the target and the TMB solutions, followed by electrochemical readout using square-wave voltammetry, which lasts only 90 min in the presence of TMB as a redox marker.

We first examined several redox markers (TMB, MB, and Sybr Green I—data not shown) and we chose TMB since it yielded the highest current-to-blank ratio. They are usually used as intercalation agents to reveal DNA hybridization, although TMB is less used than MB and Sybr Green I is used for fluorescence. After that, the main parameters influencing the readout signal such as the pIL6 concentration, its incubation time, tIL6 hybridization time, the TMB concentration, etc. were optimized using the amperometric signal and signal-to-blank ratio as selection criteria for double precision.

Under optimal conditions, the tIL6 target can be detected in less than 90 min. Using spiked buffer solutions with the target sequence, the calibration curve of the bioassay spreads over six decades of concentrations (from 10 fM to 10^7^ fM) with a detection limit as low as 7.3 fM. Furthermore, the bioassay had a high specificity since it showed a weak amperometric response when the bioconjugate was challenged with the major allele, and two mismatched and noncomplementary sequences. Using 10–fold higher concentrations of the interfering sequences, lower responses (less than 25%) were observed, which were merely that obtained response with the blank, showing a high specificity of the bioassay. A good shelf life of nine weeks was also observed when the pIL6/MBs response was assayed periodically over a period of three months.

The analysis of 23 real samples of genomic DNA voluntarily collected from 6 controls and 17 cancer patients revealed that the bioassay can differentiate between the controls having the major allele and the patients which have one or two point mutations in one or two alleles of the IL6 gene. A good discrimination between the samples was clearly observed. Furthermore, statistical analysis between the three sets of samples each time provided a *p*-value less than 0.001, denoting that the differences can be considered statistically highly significant.

The designed bioassay has several key advantages such as: (i) use of commercial reagents readily available on the market thus avoiding the cumbersome preparation and functionalization of the nanomaterials, (ii) rapidity of the testing that lasts less than 90 min and can be shortened, (iii) non-sensitivity to mismatched and noncomplementary DNA, and (iv) having a large dynamic range and a low detection limit. The bioassay can be virtually adapted to the analysis of any genomic SNP samples in monoplexed or multiplexed modes using 20–30 bp detection probes since it can distinguish between the mutated and wild DNA sequences. Further automation could yield better precision and expend the dynamic range and lower the detection limit since it will reduce the error due to the human manipulation.

## 5. Conclusions

An easy-to-implant, specific, and highly sensitive bioassay to detect SNPs in human DNA is reported using readily available reagents and can be carried out in less than 90 min. Under optimal conditions, the bioassay has a dynamic range from 10 fM to 10^7^ fM with a low detection limit (7.3 fM). Furthermore, the bioassay can differentiate heterozygous and homozygous patients from the control subjects, which was confirmed by statistical analysis. The main features of the biosensor, such as cost effectiveness, scalability, selectivity, rapidity, and ability to discriminate the mutated allele from the ancestral one, make it prone to applicability in the field of biomedical analysis of DNA genetic variations. The bioassay is easily adaptable to other SNPs and can be multiplexed to simultaneously detect several point mutations in one bioassay.

## Figures and Tables

**Table 1:** Sequences of the DNA oligonucleotides used in this work. The point mutations are underlined.

Designations	Sequences (5′ to 3′)
pIL6-bt	Biotin-AAAATAGTCTTGTACCATTTCTCTC
tIL6	GAGAGAAATGGTACAAGACTA
tIL6-1m	GAGAGAAATGTTACAAGACTA
tIL6-2m	GAGAGAAATGTTACAATACTA
Noncomplementary	GAGGACGTGGGCCGCCGCCTG

**Table 2:** Examined ranges influencing the analytical signal and the selected optimal values for this work.

Parameters	Ranges	Selected Values
Redox probe	TMB or MB	TMB
pIL6 concentration	0.05 µM to 1.00 µM	1.00 µM
TMB dilution factor	1× to 12×	3×
pIL6 reaction time	10 min to 60 min	30 min
tIL6 hybridization time	15 min to 60 min	45 min
TMB intercalation time	5 min to 20 min	15 min

**Table 3:** Selected set of electrochemical DNA sensors for detecting mutations in DNA.

Electrodes	Enzyme	Gene	Technique	Dynamic Range (fM)	Detection Limit (fM)	Real Sample	Ref.
tDNA-invertase/pDNA/MBs	GOx	NR ^a^	i-t	1 × 10^5^–1 × 10^8^	1 × 10^5^	NT ^b^	[42]
tDNA/cDNA/pDNA/MBs/SPCE	ALP	P53	DPV	1 × 10^4^–2 × 10^6^	5.9 × 10^3^	PCR amplicon	[43]
tDNA/cDNA/rDNA/MBs/SPCE	ALP	PCA3	i-t	5 × 10^3^–1 × 10^5^	5 × 10^3^	NT	[44]
tDNA/DIG-DNA-Bt/Strep/AuEA	HRP	16S rNA	i-t	1 × 10^1^–1 × 10^7^	10	Genomic DNA	[45]
tDNA/pDNA-MBs/TTF-Au/SPEs	HRP	lacZ	i-t	5 × 10^−3^–2.3 × 10^−2^	4.9 × 10^−3^	PCR amplicon	[46]
tDNA/hairpin-pDNA/AuNPs/SPCE	No	IL6 TGFβ1	DPV	1 × 10^2^–1 × 10^8^ 5 × 10^1^–1 × 10^5^	47.9 fM 16.6 fM	Genomic DNA	[39]
zipDNA/StrepMBs/SPCE	No	F5	DPV	1.5 × 10^6^–7 × 10^6^	1.62 × 10^6^	PCR amplicon	[47]
tDNA/pDNA/CM/GQDs/ITO	No	APOE4	DPV	2 × 10^7^–4 × 10^8^	4.8 × 10^5^	NT	[38]
tDNA/pDNA/spaNQ/ PAMAM/PPy/Fe3O4	No	rpoB	SWV	1 × 10^0^–1 × 10^2^	4.0	PCR amplicon	[23]
tDNA/pDNA/ StrepMBs/SPCE	No	IL6	SWV	1 × 10^1^–1 × 10^7^	7.3	Genomic DNA	This work

^a^: not reported; ^b^: not tested; AuNPs: gold nanoparticles; AuEA: gold electrode array; CM: curcumin; DIG: digoxigenin; Bt: biotin; cDNA: complementary DNA; tDNA: target DNA; pDNA: probe DNA; GQDs: graphene quantum dots; ITO: indium-doped tin oxide; PPy: polypyrrole; spaNQ: thiolated naphthoquinone; SPCE: screen-printed carbon electrode; TT: tetrathiafulvalene.

## Data Availability

The data presented in this study are available on request from the corresponding author.
